# Disease-Related Risk Factors for Caregiver Burden among Family Caregivers of Persons with Schizophrenia: A Systematic Review and Meta-Analysis

**DOI:** 10.3390/ijerph19031862

**Published:** 2022-02-07

**Authors:** Man-Man Peng, Jianli Xing, Xinfeng Tang, Qinglu Wu, Dannuo Wei, Mao-Sheng Ran

**Affiliations:** 1 Institute of Advanced Studies in Humanities and Social Sciences, Beijing Normal University at Zhuhai, Zhuhai 519087, China; wuqinglu@bnu.edu.cn; 2 Department of Social Work and Social Administration, University of Hong Kong, Hong Kong, China; u3005458@connect.hku.hk (J.X.); danorw@connect.hku.hk (D.W.); msran@hku.hk (M.-S.R.); 3 Department of Psychology, Renmin University of China, Beijing 100872, China; tang.xinfeng@foxmail.com

**Keywords:** caregiver, burden, schizophrenia, family caregiving, risk factors, meta-analysis

## Abstract

This study aimed to conduct a quantitative synthesis of the clinical correlates of caregiver burden in schizophrenia studies published in the last two decades. Derived from eight electronic databases, this meta-analytic review revisits 34 English articles published from 2000 to 2020 relevant to family caregiver burden in the schizophrenia field. The Newcastle–Ottawa Scale (NOS) was used to assess study quality. The pooled effect sizes of the selected studies ranged from −0.390 to 0.751. The results indicated a significant association between a heavier burden and disease-related risk factors, including more severe symptoms, greater general psychopathology, greater severity of functional impairment, and longer duration of illness. The results show moderating effects of study characteristics (i.e., study quality, participants, and location) on the correlations between these disease-related risk factors and caregiver burden. This review highlights the roles of study characteristics in affecting the inconsistent results for the effects of disease-related risk factors on caregiver burden in families of patients with schizophrenia. Psychosocial interventions are essential for family caregivers of persons with schizophrenia. Future studies incorporating random samples from both high-income and low-to-middle-income countries will be crucial to understand the effects of cultural contexts on caregiver burden in families of persons with schizophrenia.

## 1. Introduction

Schizophrenia is a chronic psychiatric disorder accompanied by disruptions in perception, cognition, language, emotions, behaviors, and life-related functioning [1,2]. As one of the most debilitating and costly mental disorders, schizophrenia affects approximately 7 per 1000 persons aged from 15 to 35 years [1]. According to epidemiological statistics [3], the lifetime morbid risk for schizophrenia is 7.2 per 1000 people at the median level. Family is an important extension of the mental health system; thus, family caregivers (FCGs) often play an irreplaceable role in taking care of persons with schizophrenia (PwSs) in many societies [4]. Schizophrenia requires comprehensive and multidimensional care and thus the individual and the family might be engaged in long-term care across the illness trajectory [5]. Substantial evidence has emerged suggesting that the effects and burdens of schizophrenia extend beyond PwSs to FCGs [6,7]. FCGs are confronted with a heavy burden, especially in undeveloped areas where shortages of medical resources and public health services are more common [8,9,10].

Caregiver burden is defined as an interaction between a caregiver and the surroundings (including the demands of caregiving) that is assessed by the caregiver as surpassing his/her available resources and being a threat to personal wellbeing [11,12]. In previous studies of schizophrenia caregiving, caregiving burden is categorized into objective and subjective burden [12,13]. Objective burden includes the realistic impacts of the illness on a caregiver, such as economic losses, family conflicts, and reduced time for entertainment or social activities [14,15,16]. Subjective burden refers to a caregiver’s perceived psychological distress and emotional pressure during the process of undertaking caregiving affairs, such as perception of guilt, sense of disgrace or embarrassment, and feelings of being disdained by others [15,17,18]. In the present meta-analytic study, caregiver burden refers to both subjective and objective burdens perceived by the FCGs of PwSs. The widely used measurement tools for assessing caregiver burden include the Zarit Burden Interview (ZBI) [19], the Family Burden Interview Schedule (FBI) [20], and the Burden Assessment Scale (BAS) [21], etc.

The commonly used diagnostic classification systems for schizophrenia include the International Classification of Diseases (ICD) [22] and the Diagnostic Statistical Manual (DSM) [23]. Diagnostic criteria of schizophrenia in the ICD have two differences from that in the DSM. One is that functional impairment is taken into account as a diagnostic criterion in the DSM but is not included in the ICD [24]. Another difference is the duration of psychotic symptoms to make the diagnosis of schizophrenia. In the ICD, psychotic symptoms should last for more than one month; the DSM mentions that the psychotic symptoms together with prodromal or residual symptoms should be present for at least six months [25]. According to the DSM, functional impairment is one of the clinical features and markers of schizophrenia [26,27]. Functional impairment of schizophrenia is defined as a disability in multiple aspects of daily life, including dysfunction in maintaining social relationships, sustaining occupation, and living independently [28]. Empirical evidence has shown that the caregiving burden remains high among those providing care for PwSs with a lower functioning level [29]. FCGs have an increased burden when caring for PwSs with greater severity of schizophrenic symptoms [18,30] or a longer duration of illness [31]. Nevertheless, little is known about which disease-related risk factor in PwSs has the greatest contribution to caregiver burden in FCGs, which might be targeted in future psychosocial interventions.

In the present study, symptom severity refers to the overall scores of positive symptoms, negative symptoms, and general psychopathology. Positive symptoms of schizophrenia are defined as exaggerations or loss of normal function, such as delusions, suspiciousness, and hostility [32]. Negative symptoms of schizophrenia refer to the behaviors related to the expression of emotion (e.g., blunted affect and poverty of speech) and internal experience (e.g., apathy, asociality, and anhedonia) [33]. General psychopathology of schizophrenia refers to thought disorders such as anxiety, depression, and poor attention [34]. In previous studies, inconsistent results were reported regarding the relationship between caregiver burden and specific symptom types measured using the Positive and Negative Syndrome Scale (PANSS), which includes three dimensions: positive symptoms, negative symptoms, and general psychopathology. For instance, previous studies on determinants of caregiver burden reported a significant predictive effect of the positive symptoms of PwSs on caregiver burden, especially when the caregivers needed to cope with more symptomatic behaviors such as violent behaviors [35]. Positive symptoms of PwSs, such as more bizarre and problematic behaviors, may prompt FCGs to take action, such as caring for PwSs, sending care recipients to medical appointments, or coping with troubles—which may lead to disruption of the daily lives of FCGs and greater psychological pressure [36,37]. Nevertheless, multiple empirical studies have revealed that FCGs are more likely to experience greater distress when faced with PwSs’ negative symptoms or general psychopathology but not positive symptoms [15,18]. Some studies found that caregiver burden was significantly related to the coexistence of prominent positive and negative symptoms of schizophrenia [38,39]. In other existing studies, surprisingly, positive and negative symptoms and general psychopathology of PwSs were not associated with caregiver burden [40,41,42]. However, few meta-analytic studies have been conducted to explore study characteristics that may contribute to the differences in the relationship between these disease-related risk factors (e.g., negative or positive symptoms, etc.) and caregiver burden reported by studies conducted in various countries.

In high-income countries (HICs), with the gradual implementation of deinstitutionalization and community-oriented treatment, less than 50% of PwSs live with their families [4]. However, the situation is quite different in low- to middle-income countries (LMICs). Over 70% of PwSs depend on their families in Asian countries [2,16]. The corresponding rate is even higher in China. More than 90% of PwSs are estimated to receive care from their families at home in China [9,43,44]. With distinct cultural backgrounds and sociopolitical environments, the generalization of family caregiving in patients with mental disorders is different between HICs and LMICs. For instance, in LMICs, social welfare policies and public services addressing mental health care are still underdeveloped [45]. Meanwhile, combinations of local hospitals and community-based rehabilitation services have not yet been established systematically [45,46]. Because community-based care is poorly developed in LMICs, insufficient mental health services have limited the options of rehabilitative care for PwSs [47]. Mental health services in HICs, such as Germany, tend to be more community-based strategies and are mostly provided by professionals, which thereby alleviates the caregiver burden for PwSs [48,49]. Cultural differences also increase the difficulty of identifying risk factors in patients with schizophrenia for caregiver burden across various countries. For instance, unlike individualism in Western societies, familialism, collectivism, and filial piety are important cultural values in Asian countries—such as China and India—which fundamentally promote interdependence, cooperation, and care for family members with schizophrenia [17,50,51]. Chinese and Indian families therefore prefer home care to professional organizations, and FCGs are far more involved in caring for PwSs [9,52]. However, currently, little is known about whether the study location and cultural communities affect the results regarding the effects of clinical factors of patients with schizophrenia on caregiver burden.

The objectives of this study were to conduct a quantitative synthesis of the clinical correlates of caregiver burden among FCGs of PwSs published in the last two decades. In this study, caregiver burden is defined as an interaction between an FCG and the surroundings that is assessed by the FCG as surpassing his or her available resources and being a threat to personal wellbeing [11,12]. This meta-analysis aimed to address the following three research questions: (a) What disease-related risk factors in PwSs are associated with caregiver burden in the process of providing care to PwSs? (b) What are the differences in these factors affecting caregiver burden among families of PwSs? (c) What are the moderators contributing to the differences in the associations between these factors and caregiver burden? The findings of this meta-analysis are crucial for the future development of psychosocial interventions and social support programs for FCGs of PwSs.

## 2. Methods

### 2.1. Data Sources and Search Strategy

In this meta-analysis, English-language articles were derived from the following multidisciplinary electronic databases: Web of Science, Scopus, PsycINFO, PubMed, Cochrane Library, Excerpta Medica Database (EMBASE), Nursing and Allied Health Literature (CINAHL), and Medical Literature Analysis and Retrieval (MEDLINE) database. The keywords used to identify relevant publications from these databases were (carer or caregiver or famil* or relative or spous* or parent* or child* or sibling) AND (caregiving or care or caring or nursing) AND (burden or stress or pressure or strain or distress) AND (schizophrenia or schizophrenic). The keywords were obtained based on previous systematic reviews in the study field of schizophrenia caregiving [2,6,53]. The search and selection procedure was conducted following the Preferred Reporting Items for Systematic reviews and Meta-Analyses (PRISMA) guidelines [54] (see supplementary materials Table S3). Articles published from January 2000 to February 2020 were considered in this meta-analysis. Additional articles relevant to the research topic were manually traced and checked in the lists of references in the articles. The major retrieval process was completed in March 2020.

**Figure 1:**
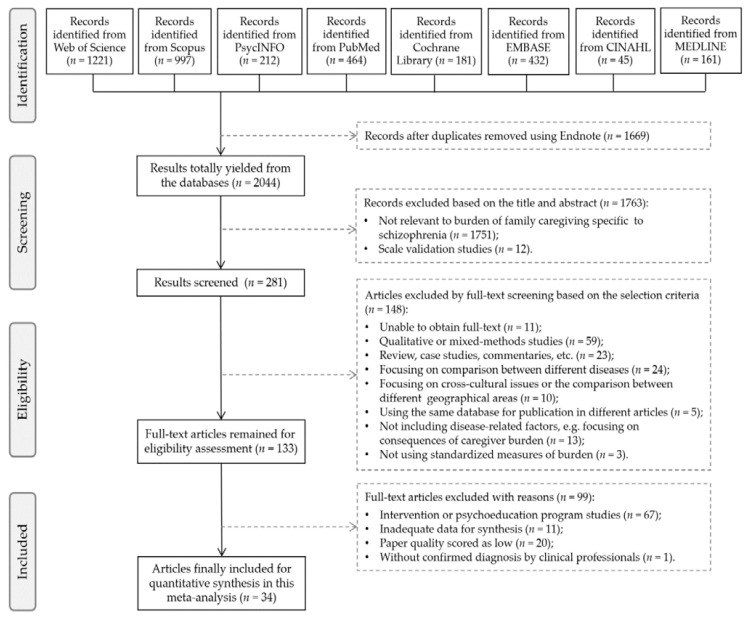
Flowchart of the selection and inclusion procedure.

Figure 1 displays the search and selection procedure of the present meta-analytic study. Initially, 3713 records were retrieved from the online databases and additional manual searches. After removing 1669 duplicates using Endnote software, 2044 records were obtained from the databases. After screening the titles and the content of abstracts, 1763 records were excluded because they were irrelevant to the theme or they focused on scale validation. Then, 148 articles were removed after a full-text screen based on the exclusion criteria. After the eligibility assessment, 99 full-text articles were further excluded. Finally, 34 journal articles were included for quantitative synthesis in this meta-analysis. The title and abstract of each article were screened by two researchers (M.-M.P. and J.X.) independently to avoid bias and ensure reliability. Full-text articles were also retrieved and reviewed according to the eligibility criteria.

### 2.2. Eligibility Criteria

The studies were eligible for inclusion if (a) they mainly focused on any types of factors influencing caregiving burden/strain/stress among relatives who care for PwSs; (b) they were empirical studies; and (c) they had at least 30 participants in terms of the sample size. Quantitative studies were included, including cross-sectional or longitudinal designs. Only journal articles published in the English language were included. When the same database was used for publication in different articles, the study with a larger sample size was selected for this meta-analysis.

Articles were excluded from this meta-analysis if (a) the full text was unavailable in electronic databases or university libraries or could not be obtained by email request from the authors; (b) the publications were not peer-reviewed; (c) the patients in the study were diagnosed with diseases other than schizophrenia (comorbidity); (d) the diagnosis of schizophrenia in the study was not made by clinical professionals or using international diagnostic criteria (e.g., Diagnostic and Statistical Manual of Mental Disorders [DSM] or International Classification of Diseases [ICD]); (e) the studies focused on a comparison between different mental disorders; (f) the studies focused on cross-cultural issues or compared different geographical areas; (g) they were not empirical studies, such as literature reviews, comments, letters to editors, or case studies; or (h) the quality of the paper was scored as low, such as having a poor statistical analysis and inadequate data for synthesis.

### 2.3. Data Extraction

The main characteristics of the selected studies were extracted and synthetized into a consolidated form by one researcher (M.-M.P.). Another researcher (J.X.) randomly selected 50% of the 34 studies to perform data extraction independently. In the cases of any disagreements, the two researchers discussed the findings with a third researcher (X.T.) to make final decisions. The extracted and coded items included (a) author(s) and year of publication; (b) participants (FCGs and/or PwSs); (c) location; (d) sample size; (e) study design (cross-sectional or longitudinal); (f) measures of caregiver burden; (g) influencing factors and the measures; (h) effect size; and (i) sociodemographic characteristics of FCGs, such as the mean age and percentage of females. Using the method reported by a previous study [55], the baseline data were chosen for the longitudinal studies, and the data measured with the instrument with the best psychometric properties were used for studies in which multiple instruments were used to measure one variable. In the present study, disease-related risk factors for caregiver burden refer to the illness status or clinical characteristics of PwSs, which were cognitively evaluated or perceived by FCGs as the sources of their psychological distress [12].

### 2.4. Quality Assessment

The checklist from the Newcastle–Ottawa Scale (NOS) [56,57] was used to conduct the quality assessment among the selected studies (see supplementary materials Table S1). The NOS has been widely used to assess the quality of cross-sectional and cohort studies among previous reviews in the areas of mental health [58,59,60,61]. The adapted version of the NOS [62] was used to rate the quality of studies included in this review. The interrater reliability and validity of this scale have been validated [62,63]. The overall score of the adapted NOS ranges from 0 to 10, representing a low to high quality, which assigns a maximum of five points to the “Selection” (four items), two points to the “Comparability” (one item), and three points to the “Outcome” (three items) subscales [62]. Based on previous classification systems [63,64,65], the researcher categorized the quality scores into low (scored 0–4), moderate (scored 5–7), and high (scored 8–10) levels (see supplementary materials Table S2). The quality assessment was performed by two researchers (M.-M.P. and J.X.) independently. When any disagreements occurred, the two researchers discussed the findings with a third researcher (X.T.) to justify the reliability of the results. Inconsistencies of quality assessment were resolved by consensus by three researchers.

### 2.5. Analysis Procedures

We conducted a meta-analysis using Comprehensive Meta-Analysis (CMA, version 3.3, Biostat, Englewood, NJ, USA) software. First, given the between-study heterogeneity, we combined the effect size estimates using random-effect models [66]. Based on previous studies [55,66], the overall effect size was calculated through an omnibus analysis. In this review, the effect sizes of most selected studies were extracted based on Pearson’s correlation coefficients (*r*) for correlational data or calculated from other convertible statistics, such as log odds ratios for binary data and standardized mean difference for continuous data [66]. For those studies reporting only separate effect sizes of the subscales, we converted each r value into Fisher’s *Z* and then transformed the mean of Fisher’s *Z* into Pearson’s *r* as a combined effect size of the overall scale for the target variable [55,66,67]. An effect size *r* of 0.1 represents a small, 0.3 a moderate, and 0.5 a large degree of association [68]. Second, the heterogeneity test was used to measure variances among the selected studies, in which the *Q* statistic was calculated [69]. Meanwhile, the *I*^2^ value was utilized to estimate the percentage of the heterogeneity attributable to the variation, with an *I^2^* index <30% ranked as mild heterogeneity, 30% to 50% as moderate heterogeneity, and >50% as high heterogeneity [70]. The associations between influencing factors and caregiving burden were estimated by calculating pooled effect sizes with 95% confidence intervals. Third, a moderation analysis was conducted using the *Q_b_* test for categorical variables (e.g., study design, location, and study quality level) or the meta-regression method for continuous variables (e.g., publication years, female percentage, and sample size) [71]. The p value was estimated to indicate a moderating effect using the *Q_b_* test, while the *β* value was used in the meta-regression analysis. Fourth, a funnel plot was obtained to measure publication bias and test whether the selected studies were distributed symmetrically around the combined effect size [72]. Afterward, the Begg–Mazumdar rank correlation test [73] and Egger’s correlation test [74] were employed to measure the publication bias of the selected studies.

## 3. Results

### 3.1. Study Characteristics

Table 1 shows the basic characteristics of the included studies. Among the 34 selected studies, 31 studies used a cross-sectional design, and three studies used a longitudinal follow-up design. There are 10 studies being conducted in Chinese communities and 24 in non-Chinese communities (e.g., U.S., India, Italy, or Japan). The sample size of FCGs in the selected studies ranged from 51 to 709, with a mean of 170.21 (standard deviation, SD = 139.85). Among FCG participants in the included studies, the mean age ranged from 32 to 61 years, and the percentage of females ranged from 18% to 100%. Twenty-seven studies scored a moderate level of quality, and seven studies scored a high level of quality.

### 3.2. Synthesis of Different Risk Factors for Caregiver Burden

**Figure 2:**
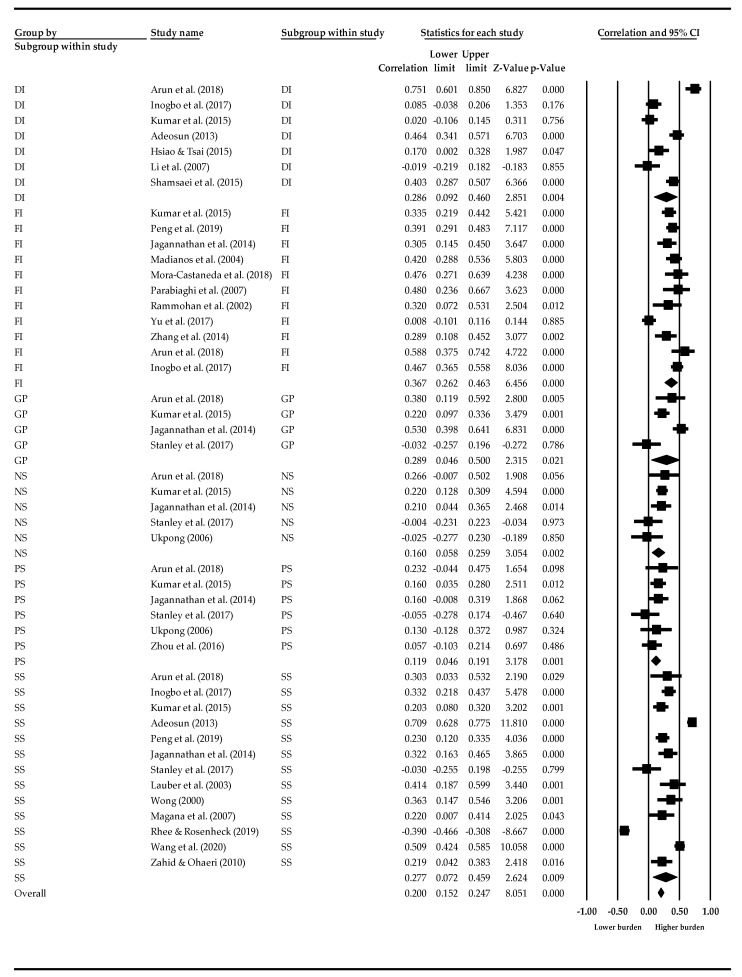
Effect sizes of clinical risk factors. SS—Symptom severity, PS—Positive symptoms, NS—Negative symptoms, GP—General psychopathology, FI—Functional impairment, DI—Duration of illness.

Table 2 shows the results of pooled effect sizes (with 95% confidence intervals) and heterogeneity tests (i.e., *Q*-value, *p* value, and *I*^2^ index) of each association between caregiver burden and the identified risk factors. The results of this meta-analysis indicated that disease-related risk factors for a heavier caregiver burden included more severe overall psychotic symptoms (*r* = 0.284, *Q* = 308.48, *p* < 0.001), greater general psychopathology (*r* = 1.107, *Q* = 19.841, *p* < 0.001), greater severity of functional impairment (*r* = 0.044, *Q* = 55.70, *p* < 0.001), and longer duration of illness (*r* = 0.295, *Q* = 77.14, *p* < 0.001). The *I^2^* statistics, ranging from 82.05 to 96.11, indicated that greater than 80% of the heterogeneity contributed to the total variation among these risk factors. Figure 2 presents the effect size of the selected studies for subgroups of disease-related risk factors. The pooled effect sizes of the selected studies ranged from −0.390 to 0.751.

### 3.3. Synthesis of Different Risk Factors for Caregiver Burden

Table 3 shows the results for the moderating effects of study characteristics on the correlations between risk factors and caregiver burden. The association between symptom severity and caregiver burden was stronger in Chinese communities than in non-Chinese communities (*Q_b_* = 31.19, *p* < 0.001). However, compared with non-Chinese communities, the associations of caregiver burden with functional impairment (*Q_b_* = 20.15, *p* < 0.001) or duration of illness (*Q_b_* = 5.96, *p* < 0.05) were significantly weaker in Chinese communities. Compared with high-income countries, studies conducted in low- and middle-income countries showed a strong association of caregiver burden with symptom severity (*Q_b_* = 164.08, *p* < 0.001).

The level of study quality exerted moderating effects on the relationship between caregiver burden and three disease-related risk factors. Our results showed a stronger association between caregiver burden and symptom severity (*Q_b_* = 21.37, *p* < 0.001) in studies with high quality than in those with moderate quality. Nevertheless, compared with studies of moderate quality, the results from high-quality studies indicated weaker associations of caregiver burden with functional impairment (*Q_b_* = 17.05, *p* < 0.001) and duration of illness (*Q_b_* = 14.63, *p* < 0.001). The associations between disease-related risk factors and caregiver burden also varied by study participant selection. Among studies that involved both FCGs and PwSs as participants, the results revealed a stronger association of caregiver burden with functional impairment (*Q_b_* = 20.15, *p* < 0.001) and duration of illness (*Q_b_* = 6.91, *p* < 0.01) than those that recruited FCGs alone. Instead, a stronger association was observed between caregiver burden and the symptom severity of PwSs (*Q_b_* = 22.26, *p* < 0.05) in the studies that recruited FCGs alone.

### 3.4. Publication Bias

The detailed results of the publication bias assessment are shown in the supplementary materials (see supplementary materials Figures S1–S6). Thirteen (for symptom severity), 6 (for positive syndrome), 5 (for negative syndrome), 11 (for functional impairment), and 7 (for duration of illness) studies were distributed symmetrically around the combined effect size. The symmetric shapes indicated no significant publication bias. The results of Egger’s test [74] and the Begg–Mazumdar rank correlation test [73] also showed no publication bias among the selected studies for symptom severity (*p*_e_ = 0.15; *p*_b_ = 0.76), positive symptoms (*p*_e_ = 0.76; *p*_b_ = 1.00), negative symptoms (*p*_e_ = 0.29; *p*_b_ = 0.81), functional impairment (*p*_e_ = 0.11; *p*_b_ = 0.64), and duration of illness (*p*_e_ = 0.19; *p*_b_ = 0.37).

## 4. Discussion

### 4.1. Main Findings

To the best of our knowledge, this meta-analytic review is the first to examine clinical risk factors for a heavier caregiver burden in FCGs of PwSs across quantitative studies conducted in different countries. In this review, the results highlight the significance of controlling the quality of studies and interview design in future meta-analytic studies aiming to explore the effects of clinical risk factors of patients with schizophrenia (i.e., symptom severity, functional impairment, and duration of illness) on caregiver burden. Furthermore, this study also discusses the differentiation of clinical risk factors on caregiver burden in schizophrenia studies between HICs and LMICs.

Consistent with previous qualitative systematic reviews [6,95], this study adds quantitative meta-analytic evidence indicating that FCGs who care for PwSs with more severe psychotic symptoms have a significantly higher risk of suffering from a heavy burden. Furthermore, in this meta-analytic study, we added to the existing knowledge that the effect size of general psychopathology on caregiver burden was larger than the overall PANSS scores. Because our findings revealed a moderating effect of study quality on the association between caregiver burden and symptom severity, we speculate that the inconsistent results regarding the effects of symptom subtypes on caregiver burden may be attributed to the varying quality of the reviewed studies [15,35,40,42]. This point deserves further investigation. The findings underscore the importance of controlling the quality of studies in future meta-analytic studies focusing on the relationship between symptom subtypes in patients with schizophrenia and caregiver burden.

Congruent with a prior qualitative review synthesizing a positive association between impaired functioning and caregiver burden [2], this study adds meta-analytic evidence to suggest that a more severe functional impairment/disability in PwSs significantly increases caregiver burden among people who provide care to PwSs. This finding highlights that families of PwSs who have a greater degree of functional impairment are more vulnerable to a heavier burden and warrant further research and service attention. The findings from this meta-analysis also indicate that FCGs caring for PwSs with longer durations of illness are more likely to experience a heavier burden than their counterparts. These findings are consistent with the broader empirical evidence reported in a recent systematic review suggesting the negative effects of a long duration of psychosis on family members of people with severe mental illness [96]. Anti-stress intervention and family support programs are essential, particularly for families with a family member suffering from a chronic type of schizophrenia.

In the present study, compared with other cultural contexts, the association between symptom severity and caregiver burden was stronger, but the association of caregiver burden with functional impairment was significantly weaker in the studies conducted in Chinese communities. We speculate that this result may be partially attributable to the social consequences of cultural-related public and self-stigma associated with psychosis syndrome, especially social isolation of the PwSs [2] and the concerns regarding ‘loss of face’ for individuals with a family member with more severe symptoms, such as symptomatic behaviors [97,98]. Another potential explanation is the cultural effect of superstition and traditional religious beliefs and limited knowledge of mental health in Chinese communities [99]. More severe symptoms, such as hallucinatory behavior, hostility, and delusions, may make people experience more fear and anxiety than the disabilities of PwSs [100,101]. Therefore, psychosocial interventions targeting FCGs of PwSs should consider the role of cultural context in psychopathology to better understand their dilemma and subjective burden. Furthermore, the associations of caregiver burden with clinical risk factors were moderated by participant selection. Specifically, compared with studies involving both FCGs and PwSs as participants, we identified a significantly stronger association between caregiver burden and the symptom severity of PwSs but weaker associations of caregiver burden with functional impairment and duration of illness in the studies recruiting FCGs alone. The presence of FCGs and PwSs at the visits in which the surveys were conducted simultaneously affects their answers to the questionnaire. As a result, future investigations on the caregiving burden of schizophrenia should carefully consider the specific design of questionnaires and interviews to reduce the external influence on the participants.

### 4.2. Strengths and Limitations

One strength of this meta-analytic study includes the strict use of PRISMA methodology [54] and a comprehensive search of eight electronic databases. Another strength is that this meta-analysis is the first to examine moderating variables of the relationships between clinical risk factors and caregiver burden, which are helpful to inform future research in schizophrenia care.

Several limitations of the present meta-analytic study must be acknowledged. First, due to the limited number of included studies and insufficient variables, the present study only measures FCG burden as a whole without differentiating the subtypes. However, given the different mechanisms of intervention and coping strategies targeted at objective burden and perceived distress [102,103], subjective and objective types of burden should be differentiated when exploring the determinants contributing to caregiver burden associated with schizophrenia in future meta-analyses. Second, because FCGs are the main focus of the present study, the findings might not be applicable to other informal caregivers of PwSs, such as domestic workers and informal nursing workers. Third, given the present study mainly focused on the risk factors of caregiver burden instead of the impacts of chronic care, the outcomes resulting from heavy caregiving burden on both FCGs and PwSs could be considered in future meta-analytic studies. Fourth, substantial heterogeneity was observed that was not explained by the relationship of caregiver burden with positive or negative symptoms. Fifth, the present meta-analysis examines only clinical risk factors associated with caregiver burden. Future meta-analyses should include both risk and protective factors for caregiver burden beyond the scope of disease-related factors (e.g., cultural, socioeconomic factors). For instance, having additional dependent relatives and increased family demands are likely to increase caregiver burden, but having more FCGs (e.g., secondary caregivers) might be helpful to reduce the burden of caregivers for PwSs, especially in Chinese communities [16,79,104].

### 4.3. Implications for Services, Research, and Policy

Notwithstanding the limitations, the findings of this meta-analytic study have significant implications for mental health services, research, and policy. First, this study underscores the need to build and raise the awareness of health care providers and policy-makers of this vulnerable population—the FCGs of PwSs. A shortage of medical resources and limited public health services are more common in underdeveloped areas; thus, family is often a significant extension of the mental health system [8,16,42,45]. Families with a PwS suffering from chronic types of schizophrenia warrant more attention. Identifying the disease-related risk factors for a heavier caregiving burden is of great value for alleviating the burden of care in the families of PwSs, reallocating limited public health resources in communities, and solving the public health problem of schizophrenia care in many societies [105,106]. Second, to answer the first and second research question, we found that the clinical status of PwSs significantly contributing to the burden of their FCGs includes more severe symptoms in the subtype of general psychopathology, a higher degree of functional impairment, and a longer duration of illness. Previous evidence-based studies indicated that under a heavy burden of care, the regular life of FCGs is changed, and their quality of life declines [107,108]. Therefore, in addition to promoting treatment and medical assistance for PwSs, their FCGs are also vulnerable populations that should be provided with more effective social support programs and mental health services. It would be helpful to assist them in dealing with psychological distress and promoting social welfare to alleviate the burden of care [45,109].

Third, to respond to the third research question, we found that when exploring the relationships between disease-related risk factors and caregiver burden, the inconsistent results are associated with the varying quality of studies and interview design. This finding serves as a reminder for future schizophrenia care studies in terms of the significance of strictly controlling study quality and improving study design. Fourth, the present study identifies research gaps in the existing literature. Few longitudinal studies have been conducted to evaluate the predictive effects of clinical risk factors on caregiver burden during a transition in the FCGs of PwSs. Moreover, this study shows that little is known about the differences in disease-related stressors of FCGs across different cultural contexts. Future studies incorporating random samples from both HIC and LMIC populations may help researchers understand how the cultural contexts affect the differentiation of clinical risk factors for caregiver burden among FCGs of PwSs. This information would help clinicians design culturally specific antistress interventions. We therefore recommend that these topics and directions for future research receive more attention.

## 5. Conclusions

The findings of this meta-analysis indicate that Chinese communities, study quality, and study participants have moderating effects on the associations of caregiver burden with symptom severity, functional impairment, or duration of illness. The effect size of the relationship between symptom severity and caregiver burden in LMICs is higher than that in HICs. This meta-analysis highlights the roles of study characteristics in affecting the inconsistent results for the effects of disease-related risk factors of patients with schizophrenia on caregiver burden. The findings also underscore the importance of controlling study quality and the recruitment of both PwSs and FCGs in future meta-analytic studies focusing on the relationship between disease-related risk factors of patients with schizophrenia and caregiver burden.

## Figures and Tables

**Table 1:** Characteristics of the included studies (*n* = 34).

Author(s) (Year)	Study Design	Sample Size		Location	Chinese	CGs Demographics		Measure of Caregiver Burden	Quality Score
		PwSs	FCGs			Mean Age	% of Females		
Wang et al. (2020) [30]	CS	-	324	China	Yes	45.19	64.8%	CBI	9
Yu et al. (2019) [51]	CS	-	355	China	Yes	32.33	45.9%	FBIS	9
Rhee & Rosenheck (2019) [75]	LS	446	446	U.S.	No	52.8	73.1%	FEIS	5
Peng et al. (2019) [37]	CS	300	300	China	Yes	58.4	50.3%	BSFC-s	8
Mora-Castañeda et al. (2018) [76]	CS	70	70	Colombia	No	60.1	74%	ZBI	6
Arun et al. (2018) [31]	CS	52	52	India	No	42.4	42.3%	BAS	6
Yu et al. (2017) [16]	CS	-	327	China	Yes	57.68	54.0%	FBIS	8
Villalobos et al. (2017) [77]	LS	60	60	U.S.	No	55.12	81.7%	BAS	5
Stanley et al. (2017) [40]	CS	-	75	India	No	49.36	37.24%	ZBI	6
Inogbo et al. (2017) [29]	CS	255	255	Nigeria	No	45.1	65.5%	ZBI	5
Zhou et al. (2016) [42]	CS	243	152	China	Yes	47	17.8%	FEIS	6
Shamsaei et al. (2015) [78]	CS	-	225	Iran	No	53.3	73.7%	ZBI	6
Kumar et al. (2015) [15]	CS	-	245	India	No	44.1	52.7%	BAS	8
Hsiao & Tsai (2015) [79]	CS	-	137	Taiwan, China	Yes	41.44	53.3%	CBS-B	6
Zhang et al. (2014) [80]	CS	110	110	China	Yes	NR	NR	FBIS	7
Jagannathan et al. (2014) [18]	CS	137	137	India	No	49	51.1%	BAS	7
Durmaz & Okanli (2014) [81]	CS	62	62	Turkey	No	41.59	45.2%	ZBI	5
Kate et al. (2013) [82]	CS	100	100	India	No	45.94	44%	FBI	5
Hanzawa et al. (2013) [35]	CS	116	116	Korea	No	55.3	55.2%	ZBI	7
Adeosun (2013) [83]	CS	181	181	Nigeria	No	44.8	60.2%	ZBI	5
Igberase et al. (2012) [13]	CS	-	200	Nigeria	No	50	39.4%	BQ	7
Zahid & Ohaeri (2010) [84]	CS	-	121	Arabian gulf	No	39.8	33.9%	IEQ	7
Hanzawa et al. (2008) [85]	CS	-	57	Japan	No	NR	100%	ZBI	6
Parabiaghi et al. (2007) [86]	LS	-	51	Italy	No	51.5	55%	IEQ-EU	5
Magaña et al. (2007) [87]	CS	85	85	U.S.	No	55.1	85.0%	ZBI	6
Li et al. (2007) [88]	CS	-	96	China	Yes	32.9	54.2%	CBS	6
Chien et al. (2007) [89]	CS	-	203	Hong Kong, China	Yes	45.22	55.2%	FBIS	9
Ukpong (2006) [41]	CS	-	60	Nigeria	No	52.33	60.0%	CBI	5
Madianos et al. (2004) [90]	CS	-	171	Greece	No	58.3	70.0%	FBS	7
Lauber et al. (2003) [36]	CS	-	64	Switzerland	No	61.0	33%	IMBF	6
Rammohan et al. (2002) [91]	CS	60	60	India	No	48.5	52.0%	BASS	6
Magliano et al. (2002) [92]	CS	-	709	Italy	No	57.1	71.09%	FPQ	8
MacInnes & Watson (2002) [93]	CS	-	107	U.K.	No	48.4	77.6%	CBD	6
Wong (2000) [94]	CS	-	74	Hong Kong, China	Yes	55.0	82.4%	PSS	6

Note: CS = cross-sectional study; LS = longitudinal study; NR = not reported. Burden Assessment Scale (BAS); Burden Assessment Schedule of Schizophrenia (BASS); Burden Scale for Family Caregivers–short (BSFC-s); Burden Questionnaire (BQ); Caregiver Burden Index (CBI); Caregiver Burden Dimensions (CBD); Caregiver Burden Scale (CBS); Caregiver Burden Scale-Brief (CBS-B); Family Burden Interview Schedule (FBI); Family Burden Interview Schedule–Short (FBIS-S); Family Burden Scale (FBS); Family Experience Interview Schedule (FEIS); Family Burden Questionnaire (FBQ); Involvement Evaluation Questionnaire; Involvement Evaluation Questionnaire, European Version (IEQ-EU); Interview for Measuring the Burden on the Family (IMBF); Perceived Stress Scale (PSS); Zarit Burden Interview (ZBI).

**Table 2:** Effect sizes of risk factors for caregiver burden in families of persons with schizophrenia.

Factors	k	N	Random Effect Size [95% CI]	Heterogeneity		
				*Q* Value	*p* ^a^	*I* ^2^
More severe overall symptoms	13	2359	0.284 [0.072, 0.496]	308.477	0.000	96.110
Positive syndrome	6	721	0.119 [0.046, 0.191]	4.110	0.534	0.000
Negative syndrome	5	749	0.584 [0.200, 0.968]	6.231	0.183	35.809
General psychopathology	4	509	1.107 [0.154, 2.060]	19.841	0.000	84.880
Higher degree of functional impairment	11	1778	0.044 [−0.252, 0.340]	55.702	0.000	82.047
Longer duration of illness	7	870	0.295 [0.092, 0.497]	77.140	0.000	91.566

Note: k = number of articles; N = total sample size. ^a^ two-tailed.

**Table 3:** Moderating variables of the correlations between clinical risk factors and caregiver burden.

Risk Factors	Moderator Variables	k	N	Effect Size [95% CI]	Heterogeneity			Moderator Test	
					*Q*	*p*	*I* ^2^	Total *Q_w_*	Total *Q_b_*
**PwS: Symptom severity**	**Chinese communities**							277.287 ***	31.190 ***
	Yes	3	698	0.381 [0.316, 0.443]	16.550	0.000	87.916		
	No	10	1661	0.147 [0.099, 0.194]	260.737	0.000	96.548		
	**Study quality level**							287.110 ***	21.367 ***
	High	3	869	0.335 [0.274, 0.393]	23.352	0.000	91.435		
	Moderate	10	1490	0.149 [0.098, 0.198]	263.759	0.000	96.588		
	**Study location**							144.397 ***	164.081 ***
	HIC	3	595	−0.230 [−0.305, −0.152]	58.925	0.000	96.606		
	LMIC	10	1764	0.361 [0.319, 0.401]	85.471	0.000	89.470		
	**Study participants**							286.218 ***	22.259 ***
	CG	6	903	0.335 [0.275, 0.392]	32.244	0.000	84.493		
	Dyad of CG and PwS	7	1456	0.145 [0.094, 0.196]	253.974	0.000	97.638		
**PwS: Functional impairment**	**Chinese communities**							35.550 ***	20.152 ***
	Yes	3	737	0.212 [0.142, 0.281]	26.252	0.000	92.381		
	No	8	1041	0.408 [0.356, 0.458]	9.298	0.232	24.718		
	**Study quality level**							38.649 ***	17.053 ***
	High	3	872	0.238 [0.174, 0.300]	29.165	0.000	93.142		
	Moderate	8	906	0.414 [0.358, 0.467]	9.484	0.220	26.189		
	**Study location**							52.054 ***	3.648
	HIC	2	222	0.434 [0.319, 0.535]	0.212	0.645	0.000		
	LMIC	9	1556	0.315 [0.269, 0.359]	51.843	0.000	84.569		
	**Study participants**							35.550 ***	20.152 ***
	CG	3	737	0.212 [0.142, 0.281]	26.252	0.000	92.381		
	Dyad of CG and PwS	8	1041	0.408 [0.356, 0.458]	9.298	0.232	24.718		
**PwS: Duration of illness**	**Chinese communities**							65.183 ***	5.957 *
	Yes	2	233	0.093 [−0.037, 0.220]	1.996	0.158	49.895		
	No	5	958	0.267 [0.207, 0.326]	63.188	0.000	93.670		
	**Study quality level**							56.510 ***	14.630 ***
	High	1	245	0.020 [−0.106, 0.145]	0.000	1.000	0.000		
	Moderate	6	946	0.288 [0.288, 0.346]	56.510	0.000	91.152		
	**Study location**							71.140 ***	0.000
	HIC	-	-	-	-	-	-	-	-
	LMIC	7	1191	0.235 [0.180, 0.288]	71.140	0.000	91.566		
	**Study participants**							64.230 ***	6.910 **
	CG	4	703	0.173 [0.100, 0.244]	23.439	0.000	87.201		
	Dyad of CG and PwS	3	488	0.320 [0.237, 0.398]	40.791	0.000	95.097		

Note: k = number of articles; N = sample size. * *p* < 0.05, ** *p* < 0.01, *** *p* < 0.001 (two-tailed).

## Data Availability

Not applicable.

## Supplementary Materials

The following supporting information can be downloaded at: https://www.mdpi.com/article/10.3390/ijerph19031862/s1, Table S1: Newcastle-Ottawa Scale (NOS) Adapted for Cross-sectional Studies; Table S2: Results of Quality Assessment for the Included Studies; Table S3. Preferred Reporting Items for Systematic Reviews and Meta-Analyses (PRISMA) Checklist. Figure S1: Funnel Plot of Symptom Severity in the Included Studies; Figure S2: Funnel Plot of Negative Syndrome in the Included Studies; Figure S3: Funnel Plot of Positive Syndrome in the Included Studies; Figure S4: Funnel Plot of General Psychopathology in the Included Studies; Figure S5: Funnel Plot of Functional Impairment in the Included Studies; Figure S6: Funnel Plot of Duration of Illness in the Included Studies.
