# Supplementary material for: Disease-Related Risk Factors for Caregiver Burden among Family Caregivers of Persons with Schizophrenia: A Systematic Review and Meta-Analysis
Source: Int J Environ Res Public Health. 2022 Feb 7;19(3):1862. doi: 10.3390/ijerph19031862 (PMC8835439; doi:10.3390/ijerph19031862)
Supplement: Supplementary file 1 [file ijerph-19-01862-s001.zip › ijerph-1572606-supplementary.pdf]

Supplementary I. Quality Assessment Tool

Table S1. Newcastle-Ottawa Scale (NOS) Adapted for Cross-sectional Studies

Selection: (Maximum 5 stars)

1) Representativeness of the sample:

a) Truly representative of the average in the target population. * (all subjects or random sampling)
b) Somewhat representative of the average in the target population. * (non-random sampling)
c) Selected group of users.
d) No description of the sampling strategy.

2) Sample size:

a) Justified and satisfactory. *
b) Not justified.

3) Non-respondents:

a) Comparability between respondents and non-respondents characteristics is established, and the response rate is satisfactory. *
b) The response rate is unsatisfactory, or the comparability between respondents and non-respondents is unsatisfactory.
c) No description of the response rate or the characteristics of the responders and the non-responders.

4) Ascertainment of the exposure (risk factor):

a) Validated measurement tool. **
b) Non-validated measurement tool, but the tool is available or described.*
c) No description of the measurement tool.

Comparability: (Maximum 2 stars)

1) The subjects in different outcome groups are comparable, based on the study design or analysis. Confounding factors are controlled.
   a) The study controls for the most important factor (select one). *
   b) The study control for any additional factor. *

Outcome: (Maximum 3 stars)

1) Assessment of the outcome:
   a) Independent blind assessment. **
   b) Record linkage. **
   c) Self report. *
   d) No description.

2) Statistical test:
   a) The statistical test used to analyze the data is clearly described and appropriate, and the measurement of the association is presented, including confidence intervals and the probability level (p value). *
   b) The statistical test is not appropriate, not described or incomplete.

(This scale has been adapted from the Newcastle-Ottawa Quality Assessment Scale for cohort studies to perform a quality assessment of cross-sectional studies for the systematic review, "Are Healthcare Workers' Intentions to Vaccinate Related to their Knowledge, Beliefs and Attitudes? A Systematic Review".)

Supplementary II. Results of Quality Assessment

Table S2. Results of Quality Assessment for the Included Studies

	Study	Selection				Comparability	Outcome		Total quality score	Quality level
		Sampling method	Sample size justified	Non-respondents	Measures of risk factors	Confounders controlled	Measures of caregiver burden	Estimates with CIs		
		≤1	≤1	≤1	≤2	≤2	≤2	≤1		
1.	Wang et al. (2020)	1	1	1	2	2	1	1	9	High
2.	Yu W. J. , Chen, Hu, & Hu (2019)	1	1	1	2	2	1	1	9	High
3.	Rhee & Rosenheck (2019)	1	0	1	2	0	1	0	5	Moderate
4.	Peng et al. (2019)	1	0	1	2	2	1	1	8	High
5.	Mora-Castañeda et al. (2018)	1	0	1	2	0	1	1	6	Moderate
6.	Arun, Inbakamal, Tharyan, & Premkumar (2018)	0	0	0	2	2	1	1	6	Moderate
7.	Yu, Y., et al. (2017)	1	0	1	2	2	1	1	8	High
8.	Villalobos et al. (2017)	1	0	0	2	0	1	1	5	Moderate
9.	Stanley, Balakrishnan, & Ilangovan (2017)-Correlates	0	0	0	2	2	1	1	6	Moderate
10.	Inogbo, Olotu, James, & Nna (2017)	1	0	0	2	0	1	1	5	Moderate
11.	Zhou et al. (2016)	0	0	0	2	2	1	1	6	Moderate
12.	Shamsaei, Cheraghi, & Bashirian (2015)	1	0	0	1	2	1	1	6	Moderate
13.	Kumar, Suresha, Thirthalli, Arunachala, & Gangadhar (2015)	1	0	1	2	2	1	1	8	High
14.	Hsiao & Tsai (2015)	1	1	0	2	0	1	1	6	Moderate
15.	Zhang et al. (2014)	1	0	0	2	2	1	1	7	Moderate
16.	Jagannathan, Thirthalli, Hamza, Nagendra, & Gangadhar (2014)	1	0	0	2	2	1	1	7	Moderate

17.	Durmaz & Okanli (2014)	0	0	1	2	0	1	1	5	Moderate
18.	Kate, Grover, Kulhara, & Nehra (2013b)-caregiving appraisal	1	0	0	2	0	1	1	5	Moderate
19.	Hanzawa et al. (2013)	0	0	1	2	2	1	1	7	Moderate
20.	Adeosun (2013)	0	0	1	2	0	1	1	5	Moderate
21.	Igberase, Morakinyo, Lawani, James, & Omoaregba (2012)	0	0	1	2	2	1	1	7	Moderate
22.	Zahid & Ohaeri (2010)	0	0	1	2	2	1	1	7	Moderate
23.	Hanzawa, Tanaka, Inadomi, Urata, & Ohta (2008)	0	0	0	2	2	1	1	6	Moderate
24.	Parabiaghi et al. (2007)	0	0	1	2	0	1	1	5	Moderate
25.	Magaña et al. (2007)	0	0	0	2	2	1	1	6	Moderate
26.	Li, Lambert, & Lambert (2007)	1	0	0	1	2	1	1	6	Moderate
27.	Chien, Chan, & Morrissey (2007)	1	1	1	2	2	1	1	9	High
28.	Ukpong (2006)	0	0	1	2	0	1	1	5	Moderate
29.	Madianos et al. (2004)	0	0	1	2	2	1	1	7	Moderate
30.	Lauber, Eichenberger, Luginbuhl, Keller, & Rossler (2003)	0	0	0	2	2	1	1	6	Moderate
31.	Rammohan et al. (2002a)-burden & coping	0	0	0	2	2	1	1	6	Moderate
32.	Magliano et al. (2002)	1	0	1	2	2	1	1	8	High
33.	Macinnes & Watson (2002)	0	1	0	1	2	1	1	6	Moderate
34.	Wong (2000)	1	0	1	2	0	1	1	6	Moderate

Supplementary III. Funnel Plot of the Included Studies

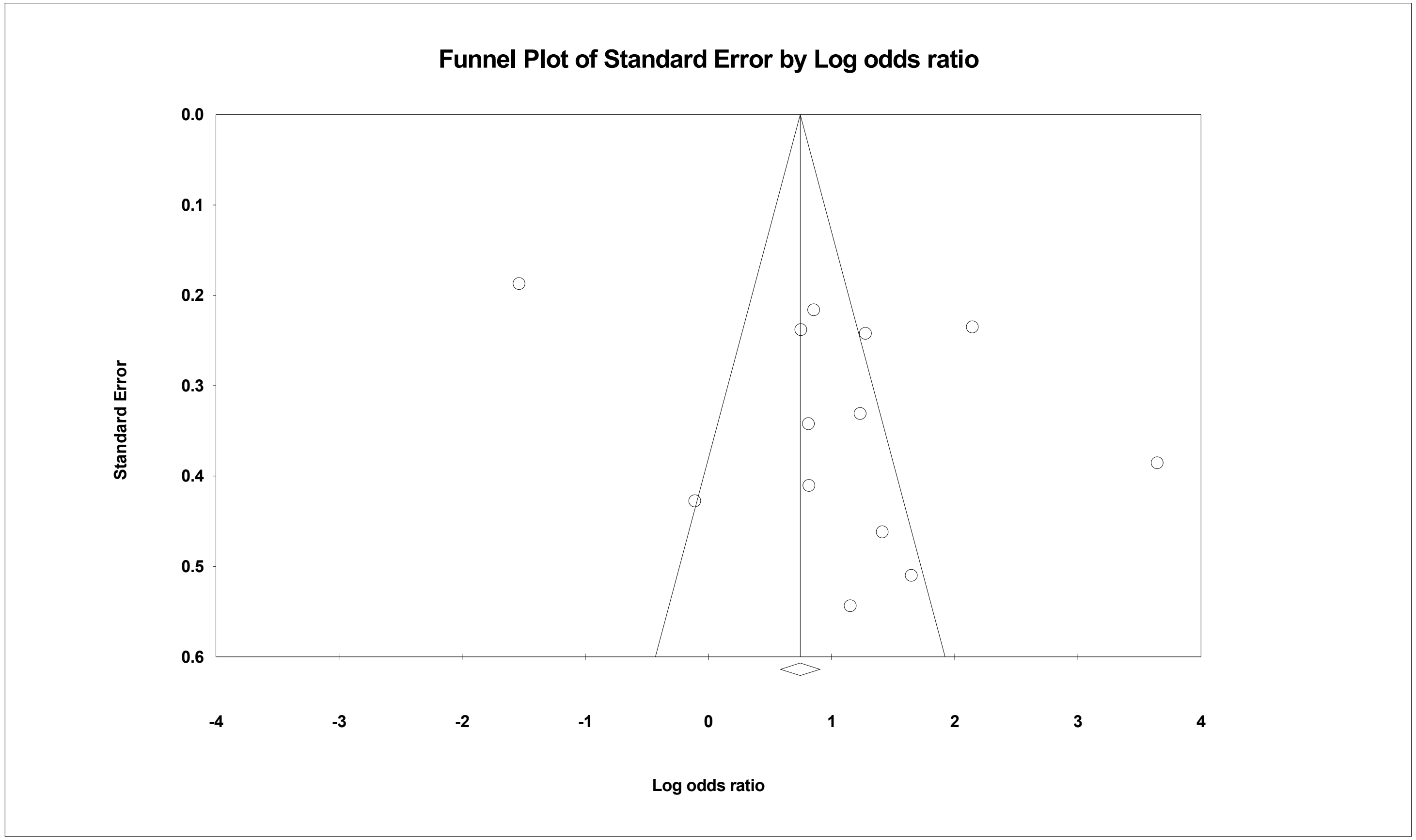

Figure S1: Funnel Plot of Symptom Severity in the Included Studies

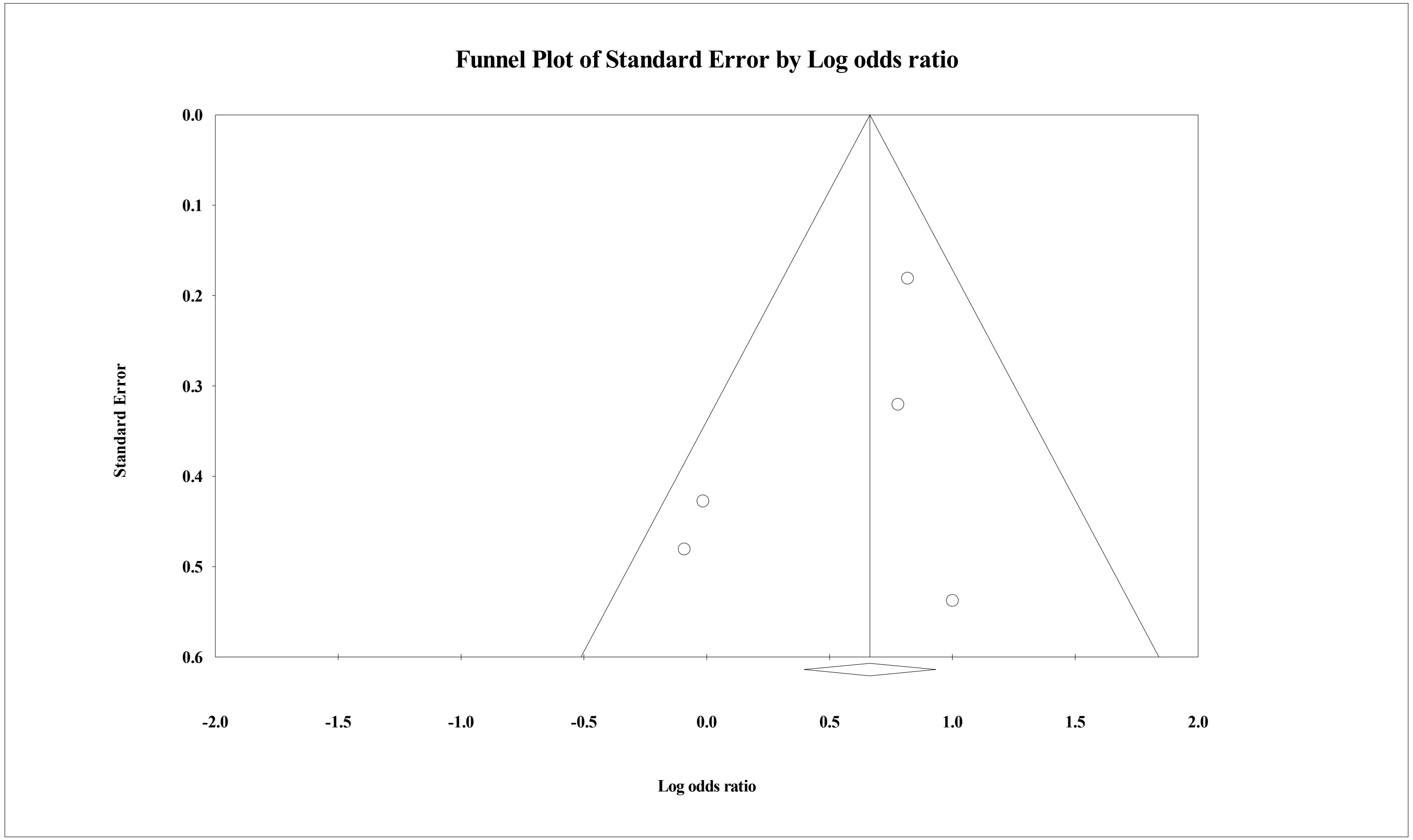

Figure S2: Funnel Plot of Negative Syndrome in the Included Studies

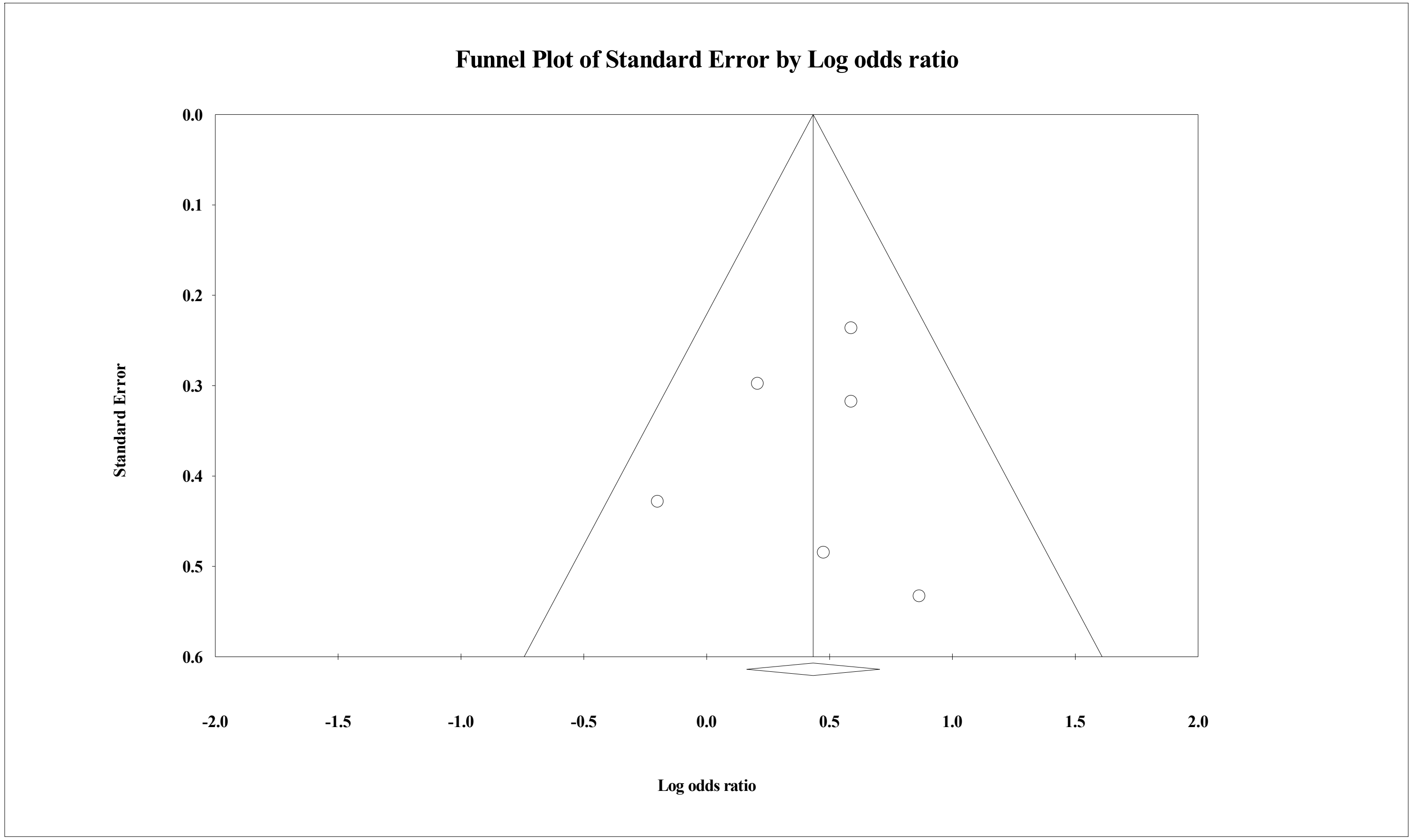

Figure S3: Funnel Plot of Positive Syndrome in the Included Studies

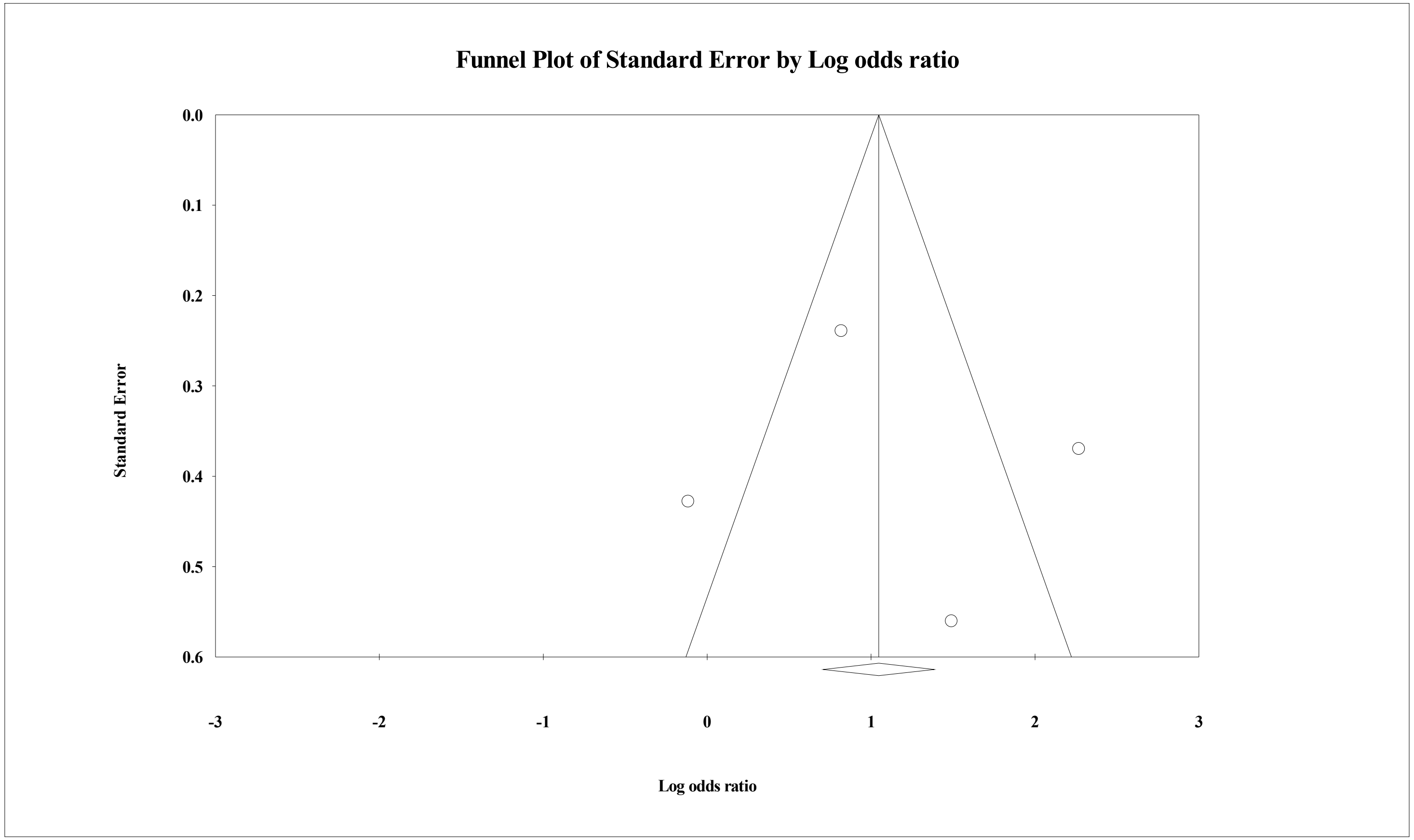

Figure S4: Funnel Plot of General Psychopathology in the Included Studies

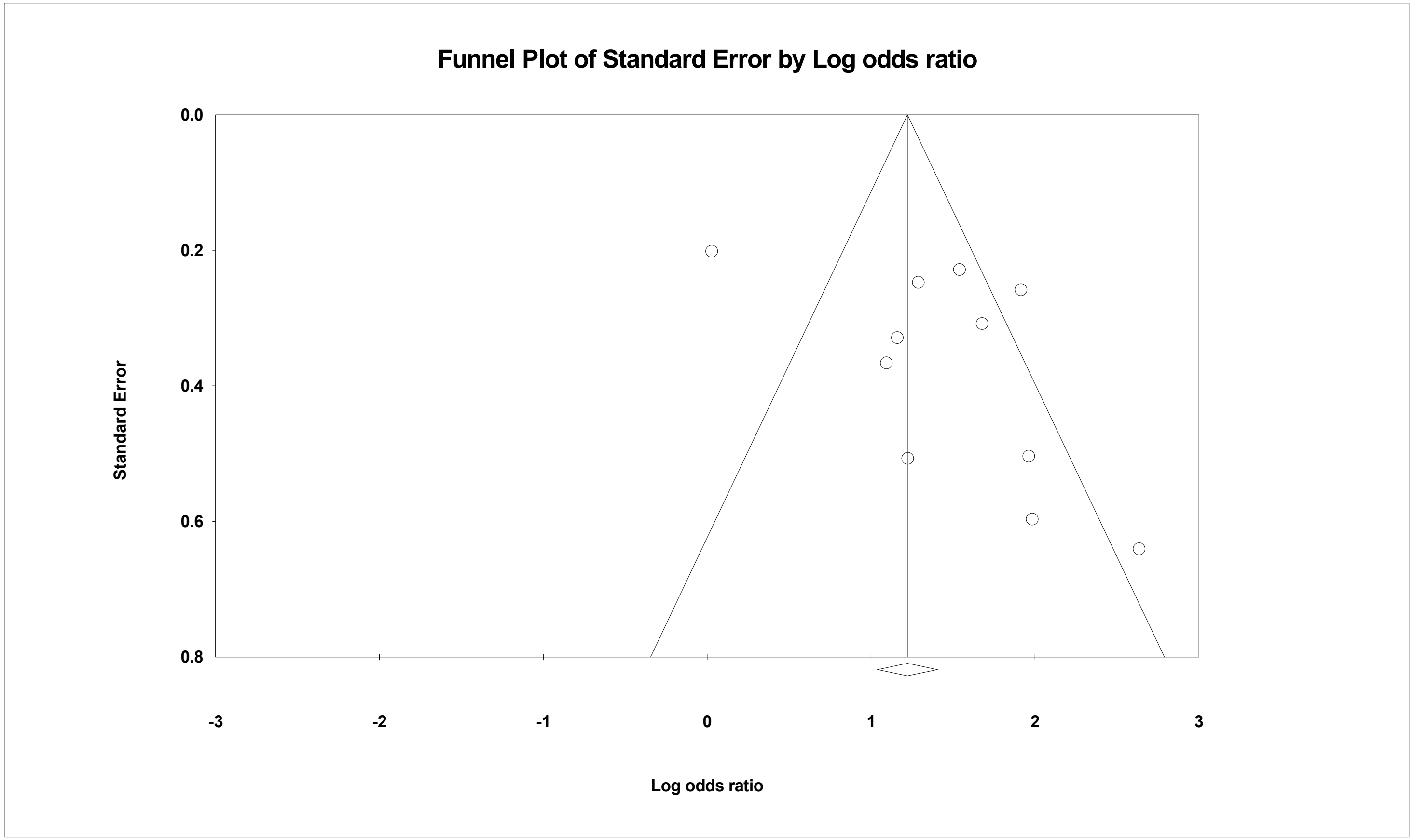

Figure S5: Funnel Plot of Functional Impairment in the Included Studies

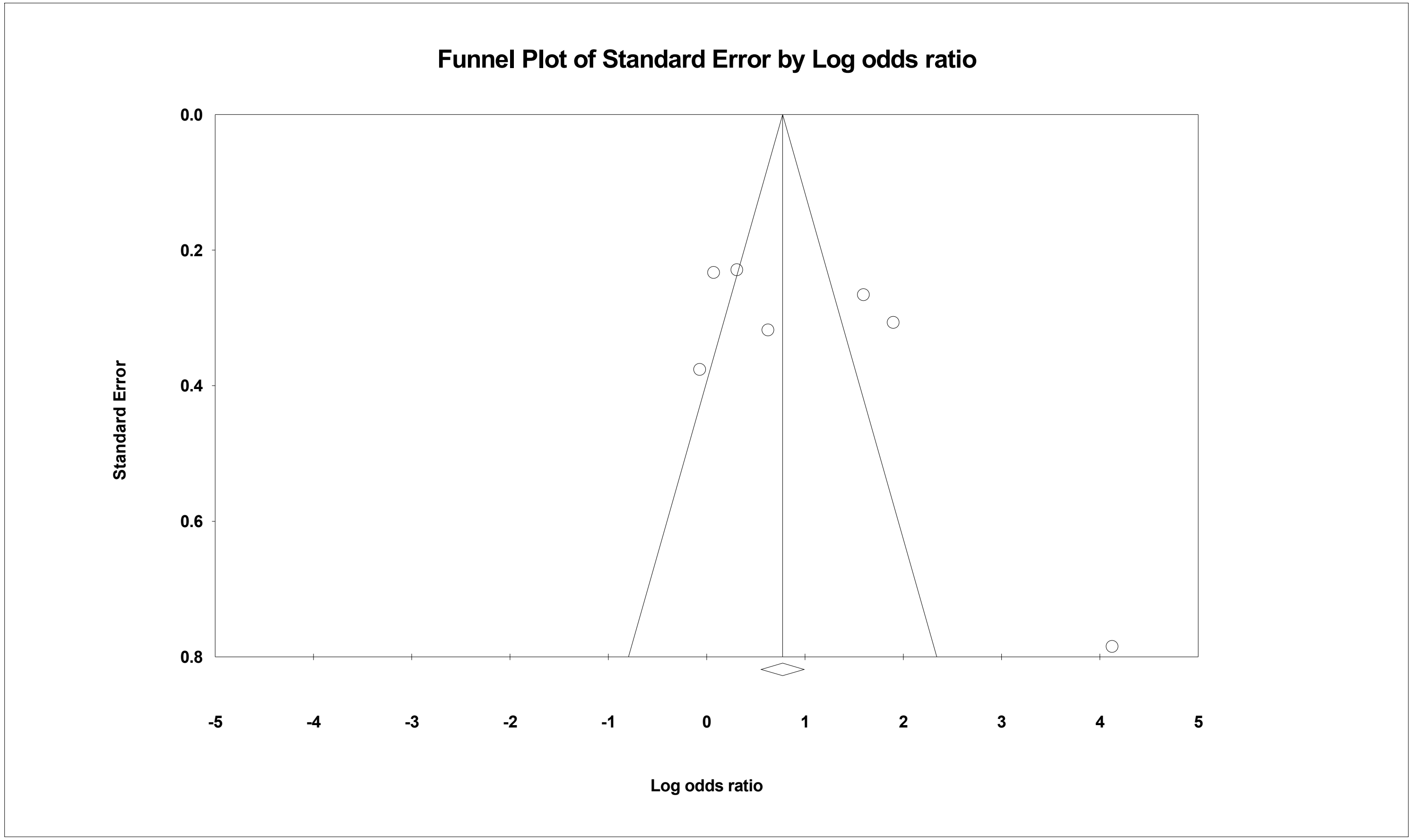

Figure S6: Funnel Plot of Duration of Illness in the Included Studies

Supplementary IV.

Table S3. Preferred Reporting Items for Systematic Reviews and Meta-Analyses (PRISMA) Checklist

Section/topic	#	Checklist item	Reported on page #
**TITLE**			
Title	1	Identify the report as a systematic review, meta-analysis, or both.	✓
**ABSTRACT**			
Structured summary	2	Provide a structured summary including, as applicable: background; objectives; data sources; study eligibility criteria, participants, and interventions; study appraisal and synthesis methods; results; limitations; conclusions and implications of key findings; systematic review registration number.	✓
**INTRODUCTION**			
Rationale	3	Describe the rationale for the review in the context of what is already known.	✓
Objectives	4	Provide an explicit statement of questions being addressed with reference to participants, interventions, comparisons, outcomes, and study design (PICOS).	✓
**METHODS**			
Protocol and registration	5	Indicate if a review protocol exists, if and where it can be accessed (e.g., Web address), and, if available, provide registration information including registration number.	✓
Eligibility criteria	6	Specify study characteristics (e.g., PICOS, length of follow-up) and report characteristics (e.g., years considered, language, publication status) used as criteria for eligibility, giving rationale.	✓
Information sources	7	Describe all information sources (e.g., databases with dates of coverage, contact with study authors to identify additional studies) in the search and date last searched.	✓
Search	8	Present full electronic search strategy for at least one database, including any limits used, such that it could be repeated.	✓

Study selection	9	State the process for selecting studies (i.e., screening, eligibility, included in systematic review, and, if applicable, included in the meta-analysis).	✓
Data collection process	10	Describe method of data extraction from reports (e.g., piloted forms, independently, in duplicate) and any processes for obtaining and confirming data from investigators.	✓
Data items	11	List and define all variables for which data were sought (e.g., PICOS, funding sources) and any assumptions and simplifications made.	✓
Risk of bias in individual studies	12	Describe methods used for assessing risk of bias of individual studies (including specification of whether this was done at the study or outcome level), and how this information is to be used in any data synthesis.	✓
Summary measures	13	State the principal summary measures (e.g., risk ratio, difference in means).	✓
Synthesis of results	14	Describe the methods of handling data and combining results of studies, if done, including measures of consistency (e.g., I^2) for each meta-analysis.	✓
Risk of bias across studies	15	Specify any assessment of risk of bias that may affect the cumulative evidence (e.g., publication bias, selective reporting within studies).	✓
Additional analyses	16	Describe methods of additional analyses (e.g., sensitivity or subgroup analyses, meta-regression), if done, indicating which were pre-specified.	✓
**RESULTS**			
Study selection	17	Give numbers of studies screened, assessed for eligibility, and included in the review, with reasons for exclusions at each stage, ideally with a flow diagram.	✓
Study characteristics	18	For each study, present characteristics for which data were extracted (e.g., study size, PICOS, follow-up period) and provide the citations.	✓
Risk of bias within studies	19	Present data on risk of bias of each study and, if available, any outcome level assessment (see item 12).	✓
Results of individual studies	20	For all outcomes considered (benefits or harms), present, for each study: (a) simple summary data for each intervention group (b) effect estimates and confidence intervals, ideally with a forest plot.	✓
Synthesis of results	21	Present results of each meta-analysis done, including confidence intervals and measures of consistency.	✓

Risk of bias across studies	22	Present results of any assessment of risk of bias across studies (see Item 15).	
Additional analysis	23	Give results of additional analyses, if done (e.g., sensitivity or subgroup analyses, meta-regression [see Item 16]).	✓
**DISCUSSION**			
Summary of evidence	24	Summarize the main findings including the strength of evidence for each main outcome; consider their relevance to key groups (e.g., healthcare providers, users, and policy makers).	✓
Limitations	25	Discuss limitations at study and outcome level (e.g., risk of bias), and at review-level (e.g., incomplete retrieval of identified research, reporting bias).	✓
Conclusions	26	Provide a general interpretation of the results in the context of other evidence, and implications for future research.	✓
**FUNDING**			
Funding	27	Describe sources of funding for the systematic review and other support (e.g., supply of data); role of funders for the systematic review.	✓

From: Moher D, Liberati A, Tetzlaff J, Altman DG, The PRISMA Group (2009). Preferred Reporting Items for Systematic Reviews and Meta-Analyses: The PRISMA Statement. PLoS Med 6(6): e1000097. doi:10.1371/journal.pmed1000097. For more information, please visit: www.prisma-statement.org.
